# Leisure and Procrastination, a Quest for Autonomy in Free Time Investments: Task Avoidance or Accomplishment?

**DOI:** 10.3389/fpsyg.2019.02918

**Published:** 2020-01-10

**Authors:** Jose Vicente Pestana, Nuria Codina, Rafael Valenzuela

**Affiliations:** Department of Social Psychology and Quantitative Psychology, University of Barcelona, Barcelona, Spain

**Keywords:** leisure, leisure time, procrastination, time budget, time investments

## Abstract

The purpose of the research was to analyze procrastination – a problem of time management that negatively affects the autonomy of people – in relation to leisure as a domain of everyday life. Specifically, the dynamics between leisure (activities and time invested – weekly frequency and duration of activity) and procrastination factors were studied. A sample of 185 university students (118 men and 67 women: *M*_*age*_ = 20.77 years, *SD*_*age*_ = 2.53) answered a procrastination scale – validated for the Spanish population – which refers to four factors of procrastination (dilatory behaviors, indecision, lack of punctuality, and lack of planning) and an adaptation of the Time Budget (TB) (a table where the participants were asked to specify “the three activities that you prefer to do when you are not studying or doing a paid job”). Results show that leisure activities are associated with factors of procrastination. As a matter of fact, the different factors of procrastination were related to specific types of leisure activities, depending on the weekly frequency of the activity or its duration. In this sense, there are cases in which the greater frequency of leisure activities (hobbies and computing, social life and entertainment) seems to contain – control, inhibit – procrastination (specifically, affecting its component of indecision) variations in the weekly frequency and duration of certain type of activities result in higher or lower scores on certain factors of procrastination. In sum, the time invested in leisure can protect from or inhibit delaying tasks – which implies enhancing the autonomy of people – a deduction that opens up new lines of research to identify optimal time investments for coping with procrastination.

## Introduction

The problem of time management known as procrastination has generated a large number of publications, due to its potential negative influence on the autonomy of people (Mouratidis et al., 2018; Won and Yu, 2018). In fact, in the educational field it has been shown that a controlling teaching style – that is, contrary to the promotion of autonomy in the students – is associated with higher levels of procrastination (Codina et al., 2018b). Even the role of leisure in task avoidance has been suggested, in the study of the relations between leisure and procrastination certain inconsistencies have been detected. These inconsistencies have to do with what is known as leisure ambivalence (Munné and Codina, 1996, 2002), given that leisure activities can have negative consequences, or on the contrary, favor personal development characterized by autonomy (Lochbaum and Jean-Noel, 2016; Balaguer et al., 2018). With the aim of discovering the dynamics of greater or less procrastination in relation to the time invested in leisure activities, the main characteristics of procrastination are described below, and later the evidence that justifies the analysis of this problem in conjunction with leisure.

As will be seen, the presence of certain leisure activities in everyday life – or the time dedicated to some of them – can affect the autonomy of people, who may see their time management negatively influenced due to their procrastination levels.

### Procrastination, a Problem of Time Management

Procrastination has been defined as the experience of time characterized by habitually – and often counterproductively – postponing the performance of tasks (Procrastination, 2019). This problem, which reveals an inefficient time management problem, difficulties or lack of motivation when it comes to the performance of certain activities in a stipulated time (Pychyl et al., 2000; Steel et al., 2018), has serious social and personal consequences (Goroshit, 2018). Expressed in numbers, this problem becomes a maladaptive lifestyle for 20–25% of healthy adults, whose autonomy can be affected (Harriott and Ferrari, 1996; Díaz-Morales and Ferrari, 2015).

Given its importance and incidence, the study of procrastination has generated a large corpus of scientific knowledge in which two main trends can be distinguished: on the one hand, the investigation of the nature of procrastination; and, on the other hand, research on the contexts and variables associated with procrastination – or which help to cope with it.

With respect to the ontology of procrastination, it has been discussed from very different perspectives, from whether it is a personality trait (Ferrari et al., 1995; Steel, 2007; Kim et al., 2017) to whether it has an intentional or irrational character (Lay, 1986; Steel, 2007; Steel and Klingsieck, 2016). Certainly, procrastination is a problem that affects people’s everyday lives; however, the assumption that procrastination is only a personality trait is a limited interpretation. Rather, procrastination appears as an individual tendency that can be influenced by certain contexts (Codina et al., 2018b). Specifically, this refers to the domains in which procrastination is most likely to occur.

With regard to the aforesaid contexts, procrastination is one of the most identified and common mistakes made in time management by students at the various different educational stages (Karatas, 2015; Cerezo et al., 2017), with academic procrastination being one of the most important domains of procrastination (Ferrari and Scher, 2000; Owens and Newbegin, 2000). In fact, 70% of college students procrastinate in their learning tasks on a regular basis (Steel and Klingsieck, 2016).

Outside the academic context, Klingsieck (2013) states that procrastination is typical of work settings, healthcare domains, everyday routines and habits, leisure, family life and partnership domains, and social life. Therefore, while procrastination has mostly been investigated in connection with work and academic behavior (Van Eerde, 2003), this does not exclude the possibility that other contexts or areas of activity have explanatory potential with respect to the phenomenon. Thus, the procrastinating tendency may be more or less intense depending on the time investment in other activities or fields of activity. For example, one may procrastinate over a professional CV update because it is more fun to spend time reading a suspense novel – even when the preparation of the CV is an activity of greater importance, urgency (or both).

### Leisure as a Context for Procrastination

The relevance of leisure has been underlined in the analysis of the uses of time made in different contexts (Roberts, 1999; Huebner and Mancini, 2003; Rojek, 2010; Kofman and Bianchi, 2012; Lam and McHale, 2015); in particular, its impact on the well-being of people has been demonstrated in different contexts (Joulain et al., 2017; Oman, 2019; Zuzanek and Hilbrecht, 2019). In fact, leisure can be a source of both well-being (and autonomy: Fattore et al., 2016) and pathological behaviors (Dorn and South, 1989; Rojek, 1999; Francis and Kentel, 2008; Codina et al., 2018a). This ambivalence – as it has been referred to by Munné and Codina (1996, 2002) – lies in the fact that leisure implies free or discretionary time that involves behaviors and experiences, which can represent a benefit, a cost, or even a mixture of the two (Kleiber et al., 2011). In our opinion, studying the relationship between leisure and procrastination helps to identify contributions concerning the above mentioned ambivalence of leisure. In this respect, an important contribution would consist of specifying what amounts of time – dedicated to a certain occupation during free time – are related to procrastinating habits or tendencies. To be precise, the explanatory potential of leisure is increased by answering a question such as the following: Is procrastination associated with certain time investments in specific types of activities, which can negatively affect the autonomy of their practitioners? In this research, time investments include two variables: frequency (times an activity is performed throughout the week) and duration (minutes used each time a given activity is performed). As we shall see, conceiving time investment in terms of frequency and duration allows the introduction of certain elucidations that can be taken into account in the joint analysis of leisure and procrastination.

On the other hand, the relations between the time invested in leisure and procrastination have been scarcely investigated in detail. Already existing approaches include highlighting associations between procrastination and reduced life satisfaction across domains like work and leisure time (Beutel et al., 2016), the different relationships between modern and postmodern values, the priority given to leisure and procrastination (Fries et al., 2005), and the suggested complex relationships between motivational interference, school-leisure conflict and procrastination (Hofer et al., 2010).

As far as time dedication is concerned, one of the characteristics of procrastinators is that they tend to structure their time to a lesser degree and tend to interchange the order in which the activities are carried out (Ferrari, 1993), while people who have routines or habits tend to procrastinate less (Steel et al., 2018). In particular, in the case of students, those who organize their academic routines within a limited time frame are less inclined to procrastinate since they plan the start of the activity and set a deadline for its accomplishment (Dietz et al., 2007; Shu and Gneezy, 2010). On the contrary, those who have a less structured time are, in a certain sense, negatively affected with respect to the autonomy that characterizes whoever is in charge of their own time.

To our understanding, the above evidence prompts a need to analyze procrastination in relation to leisure as a domain of everyday life. This analysis would make it possible to specify how much time (frequency, duration) needs to be devoted to any leisure activity to relate it to procrastination. In other words, the accuracy with respect to these time investments can make it possible to assess the degree to which a person can lose autonomy when practicing leisure – that is, given that he/she procrastinates.

### Approaching the Relationships Between Free Time Investments With Procrastination

In order to operationalize the ambivalence of leisure, the time dedicated to a leisure activity during a specific period of time has been proposed (Codina, 1999); for example, practicing sport is usually beneficial for health (U. S. Department of Health and Human Services, 2008; European Commission, 2014), but not at levels that could indicate absorption in this leisure activity in detriment to others (as in the case of sports addictions: Nogueira et al., 2018). Taking into account the operationalization of the time variable in the analysis of leisure activities, we have healthy levels when dedicating a minimum of 150 min per week, preferably spread out over 5 days (in moderate-to-intense practice: Cavill et al., 2007; Oja et al., 2010).

In order to measure time investment in leisure activities – in terms of frequency and duration – the study of leisure requires an instrument that guarantees the organization of data and flexibility when assessing it. Specifically, this research uses the technique known as the Time Budget (TB), in its behavioral-participant approach: “Behavior… is defined by the participant… on the basis of the activity in which he/she is engaged or the setting or time period in which it is embedded” (Kleiber et al., 2011, p. 58). This questionnaire is considered the most viable method for analyzing people’s daily activities, having been used by various international organizations in their studies of the use of time in different populations (Andorka, 1987; Zuzanek, 2006). In the TB, the activities of life are weighted, which allows the observation of the different degrees of significance and importance attached to different areas of life (Steinbach, 2006). In other words, thanks to the use of the TB it was possible to show that each activity has characteristics that need to be examined for their specific explanatory potential. Specifically, the TB used here recorded the activities carried out during the 7 days of the week, specifying the time spent on them, in line with the model developed by Neulinger (1986) and taking into account the adaptations and applications of TB developed for the context in which this research was carried out (Codina, 1999, 2004; Codina and Pestana, 2009; Codina et al., 2016).

As far as procrastination is concerned, its measurement must include those factors in the phenomenon that, in general, may be sensitive to different contexts. In this respect, Díaz-Morales et al. (2006) validated an instrument, which in its Spanish language version, includes the General Procrastination Scale (GP: Lay, 1986), the Decisional Procrastination Questionnaire (DP: Mann, 1982), and the Adult Inventory of Procrastination (AIP: McCown and Johnson, 1989). This instrument contemplates four factors of procrastination: dilatory behaviors (“a summary of the predisposition to manifest intention-behavior gaps”), indecision (“putting off making a decision within some specific time frame”), lack of punctuality (“inability to work diligently on a task in order to meet a deadline”), and lack of planning (“lack of self-discipline needed to stay focused on a target task”). In this instrument, procrastination is considered as a set of factors whose presence in people can affect any context of everyday life. In other words, the items in this instrument include the characteristic of procrastination mentioned above: that it is a personal tendency sensitive to different contexts and, therefore, it changes depending on the activities that a person may perform (in his/her leisure time, in the case at hand).

All told, the evidence suggests the following two hypotheses:

H_1_: Time investments (frequency and duration) in certain types of leisure activities are positively associated with procrastination factors.
H_2_: Time investments (frequency and duration) in certain types of leisure activities are positively associated with procrastination factors.

Apart from strengthening the corpus of scientific evidence regarding the relationships of procrastination with other aspects of life, specifying which leisure activities are more likely to increase procrastination levels (i.e., their factors) allows a planning and time management that would not diminish the person’s autonomy and development.

## Materials and Methods

### Design

In order to evaluate the research hypotheses, a transversal predictive design was carried out – according to taxonomy proposed by Ato et al. (2013) – in order to be able to predict which of the leisure activities (i.e., their days devoted and/or time spent in minutes) has more impact on the aforementioned procrastination factors.

### Participants

A total of 237 university students participated in the research. Cases that did not meet the required age range or presented problems in completing the instruments were ruled out. Finally, a group of 185 people – 118 men and 67 women – aged between 18 and 30 years old (*M*_*age*_ = 20.77 years, *SD*_*age*_ = 2.53) took part in the study. Participation in the study was voluntary, i.e., the participants did not receive any payment or academic benefit (such as extra marks for subjects or course credits).

### Measures

Data was collected using two instruments, accompanied by the required demographics.

#### Procrastination

The procrastination instrument was the scale validated for the Spanish population by Díaz-Morales et al. (2006) – see the whole scale in pp. 136–137). The validation in the target population of this investigation refers to four factors of procrastination (with five response options, ranging from 1 to 5, from “does not describe me at all” to “very characteristic”): dilatory behaviors, indecision, lack of punctuality, and lack of planning. Specifying procrastination in these four factors “contribute to the conceptualization of procrastination as a multidimensional, not a [*sic*] unidimensional, trait” (Díaz-Morales et al., 2006). The instrument has shown an adequate Cronbach’s alpha in this study (0.88), similar to that of the above mention validation (α = 0.83 – Díaz-Morales et al., 2006).

#### Leisure

Subsequent to the procrastination questionnaire, items about activities unaffected by academic obligations were added, following the structure and characteristics of the TB, technique described in the preceding paragraphs. Specifically, this questionnaire was presented as a table with seven columns – one for each day of the week – and a total of three rows where the participants were asked to specify “the three activities that you prefer to do when you are not studying or doing a paid job.” For each activity, the days of each week in which the activities were performed were marked, indicating the time (in minutes) dedicated to them.

### Procedure

Before collecting data, we contacted the academic office of the university whose students had been selected to take part in the sample. After obtaining the corresponding authorization to apply the instruments, discussions were held with the staff in charge of each of the groups from which information was to be collected. In all cases it was agreed to complete the instruments at 11.30 am, at the end of a 30-min morning break.

Once in the classroom, the students participated after agreeing to sign the informed consent – data confidentiality was guaranteed. The method of completing the two instruments was explained in detail. During the response process, one of the members of the research team remained in the classroom to clear up any possible doubts.

The ethical requirements of the ethics committee of the University of [^∗∗∗^Anonymized^∗∗∗^] were applied to the current study, which meant that additional approval for the research was not required since the data obtained did not involve animal or clinical experimentation. Additionally, this study complies with the recommendations of the General Council of Spanish Psychological Associations (Consejo General de Colegios de Psicólogos), the Spanish Organic Law on Data Protection (15/1999: Jefatura del Estado, 1999) and the Declaration of Helsinki (World Medical Association, 2013).

The activities accomplished during leisure time as mentioned by the participants were classified in line with the standards established by the European Union for time use surveys (EUROSTAT, 2009). These standards include ten general categories of activities: Personal care (care for own health), Employment (work done in paid jobs), Study (studies during free time), Household and family care (work done for the own household), Voluntary work and meetings (working as a volunteer free of charge or for a minor fee), Social life and entertainment (socializing with friends and relatives, as well as being a spectator/listener), Sports and outdoors activities (activities for physical exercise), Hobbies and computing (a pursuit outside regular occupation, especially for relaxation and with the computer), Mass media (reading periodicals/books, watching TV/DVD/videos, listening to the radio/records), and Travel and unspecified time (movement between two localities, activities that cannot be classified as belonging to any of the preceding groups). Of these categories, the one corresponding to Employment was not used since the TB used in this study only referred to the time of non-work. The Study category was only used in the sense of classes out of the formal education system. Also, there were no cases in which Voluntary work and meetings were carried out, so that of the ten categories only eight are included in the results analyzed.

### Analysis

For purposes of processing the data obtained, the following variables were considered: gender of the participants; time invested in leisure activities (number of days per week and time spent); and procrastination factors (dilatory behaviors, indecision, lack of punctuality, and lack of planning). As appropriate, associations between the variables were calculated using Pearson’s *r* correlation coefficient (for perceptions of time invested in leisure activities and procrastination factors). Finally, to assess the predictive effects of leisure (days devoted and time spent) on procrastination factors, regression analysis – forward method – was performed, in line with recent evidence linked to the this research (Chang, 2015; Rosly et al., 2018).

## Results

Among the leisure activities reported (Table 1), sports and outdoors activities figured prominently, mentioned by 81.6% of the participants. This activity was followed by social life and entertainment, indicated by 63.2% of the participants. Behind these two activities came mass media, mentioned by approximately one in five participants. The rest of the activities – study, household and family care, travel and unspecified time and personal care – were cited by between 2.2 and 7.6% of the total number of participants. When analyzing the activities carried out according to gender or age, significant associations were not observed.

**TABLE 1:** Leisure activities practiced: Weekly frequency and duration.

	***N***	**%**	**Frequency (days)**		**Duration (min)**	
				
			***M***	***SD***	***M***	***SD***
Personal care	4	2.2	4.75	2.87	180.00	176.63
Study	14	7.6	3.57	1.65	314.64	239.00
Household and family care	10	5.4	3.80	2.65	312.00	274.29
Social life and entertainment	117	63.2	3.98	2.09	863.38	1, 116.22
Sports and outdoor activities	151	81.6	3.96	1.62	419.43	327.02
Hobbies and computing	45	24.3	5.27	2.07	500.22	403.48
Mass media	35	18.9	5.23	1.98	439.57	353.39
Travel and unspecified time use	6	3.2	3.83	2.56	1, 200.00	1, 647.54

With regards to weekly rates for the stated activities (Table 1), hobbies and computing (*M* = 5.27, *SD* = 2.07) and mass media (*M* = 5.23, *SD* = 1.98) were reported as occurring nearly 5 days of the week. Observation of time spent doing leisure activities showed that the activities on which more minutes per week were spent – although with great disparity among the participants with regards to the time investments – were travel and unspecified time use (*M* = 1,200.00, *SD* = 1,647.54) and social life and entertainment (*M* = 863.38, *SD* = 1,116.22). Other values worth highlighting are the minutes spent on activities such as hobbies and computing (*M* = 500.22, *SD* = 403.48) and mass media (*M* = 439.57, *SD* = 353.39).

The values obtained for the four factors of procrastination (Table 2) put into manifest the predominance of dilatory behaviors (*M* = 2.77, *SD* = 0.61) and indecision (*M* = 2.63, *SD* = 0.77) was noted, being followed by the lack of planning (*M* = 2.55, *SD* = 0.56). The lack of punctuality (*M* = 2.37, *SD* = 0.86) was the factor with the lowest score (and below the midpoint). As regards values of skewness and kurtosis (idem Table 2), all factors were non-normally distributed, Regarding asymmetry and kurtosis, the four procrastination factors were distributed in a non-normal way, although the K–S test indicates a normal distribution for the factor related to dilatory behaviors.

**TABLE 2:** Means, standard deviations, skewness, kurtosis and Kolmogorov–Smirnov (K–S) test for procrastination factors.

**Procrastination**								
**factors**			**Skewness**		**Kurtosis**		**K–S**	
					
	***M***	***SD***	**Statistic**	***SE***	**Statistic**	***SE***	**Statistic**	***p***
Dilatory behaviors	2.77	0.61	0.04	0.18	0.14	0.35	0.05	0.200
Indecision	2.63	0.77	0.35	0.18	–0.04	0.35	0.11	0.000
Lack of punctuality	2.37	0.86	0.50	0.18	–0.33	0.35	0.08	0.004
Lack of planning	2.55	0.56	0.23	0.18	–0.39	0.35	0.07	0.022

The time dedicated to leisure activities – frequency and duration – brings to light other relationships that the mere realization of an activity leaves unnoticed. These relationships refer, on the one hand, to the times invested in different types of leisure activity, and, on the other hand, to the correlations between the frequency and duration of each activity and procrastination factors (Table 3).

**TABLE 3:** Intercorrelations between procrastination factors (1–4), frequency and duration of leisure activities (5–20).

	**1**	**2**	**3**	**4**	**5**	**6**	**7**	**8**	**9**	**10**	**11**	**12**	**13**	**14**	**15**	**16**	**17**	**18**	**19**	**20**
1. Dilatory behaviors	–																			
2. Indecision	0.495^∗∗^	–																		
3. Lack of punctuality	0.524^∗∗^	0.292^∗∗^	–																	
4. Lack of planning	0.603^∗∗^	0.293^∗∗^	0.416^∗∗^	–																
**Personal care**																				
5. Days devoted	–0.661	–0.062	–0.305	0.002	–															
6. Time spent (min)	–0.245	0.136	0.456	0.424	0.072	–														
**Study**																				
7. Days devoted	–0.131	0.000	0.247	0.061			–													
8. Time spent (min)	0.033	–0.035	0.862^∗∗^	0.212			0.532	–												
**Household and family care**																				
9. Days devoted	0.217	0.165	–0.007	–0.099					–											
10. Time spent (min)	0.255	0.293	–0.055	–0.125					0.829^∗∗^	–										
**Social life and entertainment**																				
11. Days devoted	–0.064	−0.202^∗^	–0.058	0.005	0.688	0.917^∗^	0.208	0.247	–0.061	0.251	–									
12. Time spent (min)	–0.123	−0.186^∗^	–0.122	–0.086	–0.101	0.276	–0.030	–0.117	–0.436	0.386	0.423^∗∗^	–								
**Sports and outdoor activities**																				
13. Days devoted	0.103	0.063	0.011	0.219^∗∗^	1.00^∗∗^	–1.00^∗∗^	–0.230	–0.738	–0.035	0.042	0.224^∗^	0.026	–							
14. Time spent (min)	0.101	0.090	0.051	0.259^∗∗^	–1.00^∗∗^	1.00^∗∗^	–0.116	–0.020	0.728	0.486	0.114	–0.061	0.607^∗∗^	–						
**Hobbies and computing**																				
15. Days devoted	–0.212	−0.292^∗^	0.032	0.066							0.289	0.086	0.129	–0.062	–					
16. Time spent (min)	–0.117	–0.244	–0.046	–0.137							0.174	0.242	–0.103	0.083	0.470^∗∗^	–				
**Mass media**																0.950^∗^	–			
17. Days devoted	–0.152	–0.142	0.037	0.024	1.00^∗∗^	–1.00^∗∗^	0.945	0.913	–1.00^∗∗^	–1.00^∗∗^	0.097	–0.007	0.086	0.163	0.987^∗∗^					
18. Time spent (min)	–0.235	–0.023	0.082	–0.141	–1.00^∗∗^	1.00^∗∗^	0.988	0.825	–1.00^∗∗^	–1.00^∗∗^	0.259	0.264	–0.073	0.151	0.968^∗∗^	0.995^∗∗^	0.637^∗∗^	–		
**Travel/unspecified time use**																				
19. Days devoted	0.038	0.119	–0.269	–0.307			–1.00^∗∗^	–1.00^∗∗^			0.818	0.232	0.497	0.570					–	
20. Time spent (min)	–0.249	–0.062	–0.549	−0.680^∗^			–1.00^∗∗^	–1.00^∗∗^			0.092	0.976^∗^	0.347	0.560			–1.00^∗∗^	–1.00^∗∗^	0.411	–

*^∗^Significant differences for a probability *p* < 0.05; ^∗∗^significant differences for significant differences for a probability *p* < 0.01.*

The significant correlations between frequency and duration of activities are understood in two ways. On the one hand, there are the correlations in which the times invested in some activities increase simultaneously, and, on the other hand, the cases in which on increasing the time investment in one activity, the investment in another decreases (either in frequency, duration or both). Among the results detailed in Table 3, four examples stand out (the first two with directly proportional correlations and the remaining two with negative correlations). Firstly, directly proportional correlations are observed between hobbies and computing and mass media as regards both frequency (*r* = 0.987, *p* < 0.001) and duration (*r* = 0.968, *p* < 0.001). Secondly, dedicating more days to personal care also increases the frequency of sports and outdoors activities (*r* = 1.000, *p* < 0.001) and mass media (*r* = 1.000, *p* < 0.001). Thirdly, the more time spent on personal care, the less is spent on sports and outdoors activities (*r* = −1.000, *p* < 0.001) and mass media (*r* = −1.000, *p* < 0.001). And fourthly, the more frequent household and family care, the less time dedicated to mass media – both as regards frequency (*r* = −1.000, *p* < 0.001) and duration (*r* = −1.000, *p* < 0.001).

In respect of the relations between procrastination factors and leisure activities, the higher the frequency of hobbies and computing (*r* = −0.292, *p* < 0.050) and social life and entertainment (*r* = −0.202, *p* < 0.050), the lower the indecision factor. A greater lack of punctuality is observed when more time is devoted to study (*r* = 0.862, *p* < 0.001). Finally, lack of planning is related to the two variables relative to time devoted to sports and outdoor activities: either in terms of frequency (*r* = 0.219, *p* < 0.001), or dedication to the activity (*r* = 0.259, *p* < 0.001).

In order to specify the explanatory potential of these correlations, multiple linear regression analyses (forward method) were carried out for each of the factors of procrastination considered (Table 4). Indecision was explained by the frequency of hobbies and computing (*R*^2^ = 0.08, *F* = 4.18, *p* < 0.047, η*_*p*_*^2^ = 0.129; Tolerance = 1.00, VIF = 1.00, Durbin-Watson value = 2.11). As regards lack of punctuality, this factor was explained by the time invested in studying (*R*^2^ = 0.74, *F* = 34,56, *p* < 0.000, η*_*p*_*^2^ = 0.902; Tolerance = 1.00, VIF = 1.00, Durbin-Watson value = 2.37). Finally, lack of planning was explained by the time invested in sports and outdoor activities (*R*^2^ = 0.06, *F* = 11.48, *p* < 0.001, η*_*p*_*^2^ = 0.411; Tolerance = 1.00, VIF = 1.00, Durbin-Watson value = 1.94). As can be seen, tests to see if the data met the assumption of collinearity indicated that multicollinearity was not a concern, as well as that of independent errors.

**TABLE 4:** Regression analysis summary for frequency and duration of leisure activities predicting procrastination factors.

**Leisure activities, models and predictor variables**	**Procrastination factors**																	
	
	**Indecision**						**Lack of punctuality**						**Lack of planning**					
			
	***B***	***SE B***	**95% CI**	**β**	***t***	***p***	***B***	***SE B***	**95% CI**	**β**	***t***	***p***	***B***	***SE B***	**95% CI**	**β**	***t***	***p***
**Study**																		
Min. in whole week							0.004	0.001	[0.002, 0.005]	0.862	5.880	0.000						
**Sports and outdoor activities**																		
Min. in whole week													0.000	0.000	[0.000, 0.001]	0.259	3.339	0.001
**Hobbies and computing**																		
Days devoted	−0.105	0.051	[−0.208, −0.002]	−0.292	−2.046	0.047												

*Each of the activities has two measures: number of days in the week in which the activities are practiced and the minutes allocated each time an activity is practiced. CI = confidence interval for B. Models based on forward method. Only the significant results are included in the table.*

## Discussion

Testing our hypotheses has shown that leisure activities are associated with factors of procrastination. These associations show that the different factors of procrastination were related to – or appear to be facilitated by – specific types of leisure activities (H_1_), depending on the weekly frequency of the activity or its duration. In this sense, there are cases in which the greater frequency of leisure activities (hobbies and computing) seems to contain – control or inhibit – procrastination (specifically, affecting its component of indecision).

This positive aspect of leisure in relation to procrastination contrasts with the duration of sports and outdoor activities, which have a directly proportional relationship with lack of planning (H_2_). This data reflects the negative side of the ambivalence of leisure [as noted, among others, by Munné and Codina (1996, 2002), Rojek (1999); Francis and Kentel (2008), and Kleiber et al. (2011)] and also the complexity of leisure itself.

Other results that should be taken into account are the relationships between time investments in different types of leisure activities, given that investing more time in a leisure activity can serve both to increase and decrease the time allocated to other activities [in line with what is evidenced by Samdahl and Jekubovich (1993); Patry et al. (2007), Hofer et al. (2009), and Grund and Fries (2012)], also potentially diminishing practitioners autonomy [which has been demonstrated by Gerber et al. (2018)]. In this sense, it is interesting to observe how personal care and sports and outdoor activities are related, since when they are valued – in terms of frequency – both types of activity correlate positively. On the other hand, when duration is valued, the greater the dedication to personal care the less time devoted to sports and outdoor activities.

It should obviously not be ignored that the presented findings are based on a specific sample of individuals – although participants’ practice of leisure activities is similar to that of more general groups with similar demographic characteristics. To be precise, our student sample is similar to that of other studies (Ministerio de Sanidad, Servicios Sociales e Igualdad, 2014; Codina et al., 2016) as regards the prevalence of sports and outdoor activities and social life and entertainment as leisure activities. Likewise, it should be noted that the measure of leisure has taken into account the activities carried out and the time allocated to them, pending – in future research – the examination of the subjective valuations and experiences linked to leisure activities (Neulinger, 1981; Kleiber et al., 1986, 2011; Lee et al., 1994; Carbonneau and Freire, 2017), especially those of linked to well-being (Joulain et al., 2017; Oman, 2019; Zuzanek and Hilbrecht, 2019).

The proper functioning of the sample data does not obviate its limitations. Although this study has had a target especially sensitive to the issue of procrastination – such as university students – future research should consider other sectors of the population with different levels of age and their specific time management problems. Likewise, a larger sample will allow making observations with regards to gender differences related to leisure activities, a reality that has been recently proven in our context (Codina et al., 2016, 2018a).

Future research should also incorporate the latest advances in the measurement of procrastination (Svartal and Steel, 2017), together with the use of the TB as an instrument for the study of leisure, since its qualitative approach provides a wide variety of distinctions that help to preclude the subjective evaluations of the researcher (Codina, 1999, 2004). In this study, this qualitative approach brought to light the participants’ perception of their use of time, but on the other hand, it impedes the characterization of time investment in activities common to most people. For example – and in the case at hand – while the low frequencies observed in habitual activities such as personal care do not imply that people do not carry them out, it does suggest that they do not have them in mind when thinking about their free time.

By specifying in more detail which leisure activities, times of the week and quantities of time are related with procrastinating behaviors, leisure could be used as a predictor variable to protect from or inhibit delaying tasks. In a more general sense, it would make sense to specify, if a person plans the activities in an important domain of his or her life, to what extent this planning not only protects against procrastination in this area but also in others, besides being able to generalize this habit to the rest of domains. Likewise, our results suggest new research perspectives that could serve to identify optimal time investments in leisure activities in order to cope with procrastination, as well as contributing with well-being and enhancing autonomy.

Put differently, taking into account the relationships between leisure and procrastination as a multidimensional construct, can be helpful to consider which free time activities – and their temporary investments – are the most appropriate to deal with procrastination (or, according to case, do not encourage it). Based on the results of this research, psychological, social and educational interventions should address time management outside formal education as a context for the development of the person in terms of their autonomy.

## Data Availability Statement

The datasets generated for this study are available on request to the corresponding author.

## Ethics Statement

All subjects gave written informed consent prior to the collection of the research data. The ethical requirements of the Ethics Committee of the University of Barcelona were applied to the current study, which meant that additional approval for the research was not required because the data obtained did not involve animal or clinical experimentation. Additionally, this study complies with the recommendations of the General Council of Spanish Psychological Associations (Consejo General de Colegios de Psicólogos), the Spanish Organic Law on Data Protection (15/1999: Jefatura del Estado, 1999), and the Declaration of Helsinki (World Medical Association, 2013).

## Author Contributions

JP conceived and designed the research and was responsible for drafting the work and revising it critically for important intellectual content. NC was responsible for the analysis and interpretation of data gathered during the research and revising it critically for important intellectual content. RV was responsible for the analysis of data gathered during the research and revising it critically for improving the explanatory potential of the results obtained.

## Conflict of Interest

The authors declare that the research was conducted in the absence of any commercial or financial relationships that could be construed as a potential conflict of interest.
